# A Cell-Based Evaluation of the Tyrosinase-Mediated Metabolic Activation of Leukoderma-Inducing Phenols, II: The Depletion of *Nrf2* Augments the Cytotoxic Effect Evoked by Tyrosinase in Melanogenic Cells

**DOI:** 10.3390/biom15010114

**Published:** 2025-01-13

**Authors:** Tomoko Nishimaki-Mogami, Shosuke Ito, Kazumasa Wakamatsu, Takumi Akiyama, Norimasa Tamehiro, Norihito Shibata

**Affiliations:** 1 Division of Biochemistry, National Institute of Health Sciences, Kawasaki-ku, Kawasaki 210-9501, Japan; tamehiro@nihs.go.jp (N.T.); n-shibata@nihs.go.jp (N.S.); 2 Institute for Melanin Chemistry, Fujita Health University, 1-98 Dengakugakubo, Kutsukake-cho, Toyoake 470-1192, Japan; sito@fujita-hu.ac.jp; 3 Division of Environmental Chemistry, National Institute of Health Sciences, Kawasaki-ku, Kawasaki 210-9501, Japan; akiyamat@nihs.go.jp

**Keywords:** chemical leukoderma, *o*-quinone, tyrosinase, Nrf2, Slc7a11, Nqo1, B16-4A5 cells, B16BL6 cells

## Abstract

Chemical leukoderma is a disorder induced by chemicals such as rhododendrol and monobenzone. These compounds possess a *p*-substituted phenol moiety and undergo oxidation into highly reactive and toxic *o*-quinone metabolites by tyrosinase. This metabolic activation plays a critical role in the development of leukoderma through the production of damage to melanocytes and immunological responses. This study aimed to develop a simple method for assessing the metabolic activation of leukoderma-inducing phenols without analyzing the metabolite. Although B16BL6 melanoma cells showed insufficient sensitivity to the cytotoxicity assay, the siRNA-mediated knockdown of the transcription factor NRF2 (NFE2L2) repressed the expression of cytoprotective factors, thereby augmenting the cytotoxicity of all six leukoderma-inducing phenols tested in a tyrosinase-dependent manner, indicating enhanced sensitivity to *o*-quinone metabolites. Additionally, the knockdown of the NRF2-target *Slc7a11* elevated the cytotoxicity of three out of the six compounds, indicating the involvement of cystine transport in cellular protection. In contrast, the knockdown or inhibition of the NRF2-target *Nqo1* had minimal effects. The same response was induced upon *Nrf2* and *Slc7a11* knockdown in B16-4A5 cells, albeit with low sensitivity owing to low tyrosinase expression. We conclude that the analysis of tyrosinase-dependent cytotoxicity in *Nrf2*-depleted B16BL6 cells may serve as a useful strategy for evaluating the metabolic activation of chemicals.

## 1. Introduction

Chemical leukoderma is a skin depigmentation disorder induced by exposure to specific chemicals including monobenzone (MB, monobenzyl ether of hydroquinone) [1], rhododendrol (RD, 4-(4-hydroxyphenyl)-2-butanol) [2,3], and raspberry ketone (RK, 4-(4-hydroxyphenyl)-2-butanone) [4]. Additionally, anti-melanoma agents such as 4-*S*-cysteaminylphenol (4SCAP) [5], *N*-propionyl-4-*S*-cysteaminylphenol (NPr4SCAP) [6,7], and environmental chemicals, including *p*-cresol (pCRE) [8] and 4-methoxyphenol [9], have been shown to induce depigmentation in experimental animal models. These chemicals possess a *p*-substituted phenol moiety and are structurally similar to tyrosine, which is a precursor of melanin [10,11]. Tyrosinase-mediated oxidation converts MB [12,13], RD [14], RK [15], 4SCAP [16], NPr4SCAP [17], pCRE [18], and a series of structurally related leukoderma-inducing phenols [19,20] into their corresponding *o*-quinone metabolites, which are highly reactive and bind to cellular glutathione, cysteine, and proteins through their cysteinyl residues, generating reactive oxygen species (ROS) [13,21,22]. It has been proposed that this metabolic activation plays a critical role in the development of leukoderma elicited by phenolic compounds through the production of melanocyte-specific damage and subsequent immune reactions [17,23].

We previously developed a cell-based assay for metabolite analysis to evaluate the susceptibility of phenolic compounds to tyrosinase-mediated metabolic activation [18]. All seven leukoderma-inducing phenols tested were oxidized to *o*-quinone metabolites in human tyrosinase-expressing 293T cells and were detected as adducts of glutathione and cysteine, whereas 2-*S*-cysteaminylphenol (2SCAP), an *o*-substituted isomer of 4SCAP, was not oxidized to detectable levels. It has been reported that 2SCAP does not act as a substrate for mushroom and mammalian tyrosinases [24]. In addition, we established an in vitro metabolite analysis method using soluble truncated human tyrosinase, which successfully identified *o*-quinone metabolites with similar substrate specificities [18]. However, metabolite analysis is a challenging task requiring the identification and quantification of novel metabolites of each phenolic compound.

The metabolic activation of phenolic compounds into *o*-quinone metabolites by tyrosinase produces cytotoxicity, as evidenced by the highly toxic chemically synthesized catechol metabolites of RD [21,25]. RD [25,26,27] and 4SCAP [18,28,29] exhibit tyrosinase-dependent cytotoxicity in specific sources of melanocytes and melanoma cells. However, the strategy of evaluating metabolic activation by detecting tyrosinase-dependent cytotoxicity is hampered by the differences in responses between cell sources. Different origins of melanocytes exhibit varying sensitivities to RD, with the reported IC_50_ values for melanocytes ranging from the micromolar to the millimolar range [26,30]. Moreover, it is difficult to obtain melanocytes with properties comparable to those previously reported in sufficient quantities. In a previous study, we, therefore, employed B16BL6 mouse melanoma cells, in which 4SCAP and MB exhibited tyrosinase-dependent cytotoxicity, whereas RD did not [18].

Several cytoprotective factors have been shown to affect cell sensitivity to leukoderma-inducing phenols. The depletion of intracellular glutathione using a selective inhibitor of glutathione synthesis resulted in increased cytotoxicity against RD in melanocytes [31]. Additionally, NF-E2-related factor 2 (NFE2L2, commonly NRF2), a master transcription factor that regulates several cytoprotective genes in response to oxidative and electrophilic stress [32,33,34], plays a protective role in melanocytes and melanoma cells, in which high levels of ROS are produced [35,36]. The knockdown of NRF2 with siRNA increases the cytotoxicity of MB [37] and RD [31] in melanocytes, whereas the constitutive activation of the KEAP1/NRF2 system decreases the cytotoxicity of MB [37] and RD [38,39]. NRF2 target genes include enzymes and transporters responsible for glutathione biosynthesis and utilization, ROS scavenging, xenobiotic metabolism, drug transport, and the thioredoxin system [32,33]. However, whether NRF2 protects against the toxicity of other leukoderma-inducing phenols, particularly against their toxic tyrosinase metabolites, remains unknown.

NAD(P)H: quinone oxidoreductase 1 (NQO1) catalyzes the two-electron reduction of endogenous and exogenous quinones [40,41,42] and is induced by NRF2 activation, which protects the cells from redox cycling and oxidative stress by detoxifying quinones [40]. The conditional overexpression of NQO1 in melanoma cells reduces RD toxicity [38]. Conversely, certain anticancer agents exert cytotoxic effects after the conversion of their quinone structure to hydroquinone by NQO1 [42,43]. However, whether NQO1 plays a protective role or potentiates the toxicity of different structural *o*-quinone metabolites of leukoderma-inducing phenols remains to be clarified.

*Slc7a11*, an NRF2-target gene, encodes a component of the glutamate–cysteine antiporter xCT [44]. xCT-mediated cystine transport is critical for maintaining cellular glutathione levels and protecting cells from oxidative stress [32,45]. A low rate of cystine transport due to a mutation in *Slc7a11* in mice results in a reduction in pheomelanin [45], which is produced by the addition of cysteine to *o*-dopaquinone. In parallel, the *o*-quinone metabolites of leukoderma-inducing phenols produced by tyrosinase bind to cellular cysteine and glutathione [17,18]. However, it remains unclear whether *Slc7a11* affects the formation of these thiol adducts and, thus, cell viability.

In this study, we developed a simple method to assess the metabolic activation of phenolic compounds by monitoring their tyrosinase-dependent cytotoxicity. As only a limited number of compounds exhibit tyrosinase-dependent cytotoxicity in B16BL6 melanoma cells [18], we employed a strategy to enhance the sensitivity of this cell line to *o*-quinone metabolites. This involved the knockdown of NRF2, a master regulator of cytoprotective genes, and the NRF2-targets *Slc7a11* and *Nqo1* [32,33,34]. The knockdown of *Nrf2* mRNA was highly effective at increasing cell sensitivity. All six leukoderma-inducing phenols tested exhibited tyrosinase-dependent cytotoxicity, whereas the knockdown of *Slc7a11* increased sensitivity to several compounds. Furthermore, increased cell sensitivity was observed in B16-4A5 cells following *Nrf2* or *Slc7a11* knockdown, which is a cell line known to produce high levels of melanin [46]. However, B16BL6 cells, which expressed higher levels of tyrosinase, exhibited greater sensitivity than B16-4A5 cells.

## 2. Materials and Methods

### 2.1. Materials

pCRE was purchased from Tokyo Chemical Industry (Tokyo, Japan); RK and MB were purchased from FUJIFILM Wako Pure Chemical Corporation (Osaka, Japan); ES936 was purchased (CAS 192820-78-3) from Santa Cruz Biotechnology, Inc. (Dallas, TX, USA); and RD was a gift from Kanebo Cosmetics Inc. (Tokyo, Japan). 4SCAP, 2SCAP, and NPr4SCAP were previously synthesized [5]. All other chemicals were of the highest purity available.

### 2.2. Cell Culture

Mouse melanoma cell lines B16BL6 and B16 melanoma 4A5 (B16-4A5) were purchased from the RIKEN BioResource Research Center (Tsukuba, Japan) and maintained in RPMI1640 medium and Dulbecco’s modified Eagle’s medium (DMEM), respectively, containing 10% FBS.

### 2.3. RNA Interference

For the siRNA knockdown experiments, siRNA was transfected into individual separate wells of a 96-well plate. B16BL6 melanoma cells (1 × 10^4^ cells/well) or B16-4A5 melanoma cells (2.4 × 10^4^ cells/well) in a 100 μL growth medium were plated directly into the transfection mixture (20 μL/well) containing the Lipofectamine RNAiMAX Reagent (0.2 μL) (Thermo Fisher Scientific, Walthem, MA, USA) and 1 pmol siRNA in the Opti-MEM Reduced Serum Medium (Thermo Fisher Scientific). The following siRNAs were utilized: Stealth RNAi™ siRNA (Thermo Fisher Scientific) for mouse *Nrf2* (*Nfe2l2*) (#1, MSS275988; #2, MSS207018), mouse *Tyr* (#1, MSS212191; #2 MSS212190), mouse *Slc7a11* (MSS218649), and mouse *Nqo1* (MSS276039), and Stealth RNAi™ siRNA negative control hi GC (Thermo Fisher Scientific). Twenty-four hours after transfection, the cells were incubated in a growth medium containing 10% FBS for an additional 24 h. Total RNA was then extracted, and the expression of each target mRNA was determined. Cell viability was determined after incubation in the medium (100 μL) containing 10% FBS and a vehicle (DMSO, 0.5%) in the presence or absence of phenolic compounds for 24 or 48 h.

### 2.4. RNA Extraction and Quantitative Real-Time RT-PCR

Total RNA was extracted from cells using the RNeasy Mini Kit with deoxyribonuclease (QIAGEN, Valencia, CA, USA). mRNA levels of various genes were determined by quantitative real-time RT-PCR. The ABI Prism 7300 sequence detection system (Thermo Fisher Scientific) and a QuantiTect Probe RT-PCR Kit (QIAGEN) were used. TaqMan probes/primers used were as follows: mouse *Tyr*, forward: 5′-AGCCTGTGCCTCCTCTAAGAACT-3′; reverse: 5′-CTTGCCGATGGCCAGAAG-3′; probe: 5′-6-FAM-TTGGCAAAA/ZEN/GAATGCTGCCCACCA-IBFQ-3′ (Integrated DNA Technologies, Coralville, IA, USA), and 18S rRNA (Applied Biosystems, Thermo Fisher Scientific), as previously described [18]. The Prime Time^®^ Std qPCR Assay primer/probe for mouse *Nrf2* (*Nef2l2*) (Hs. PT. 58.29108649), mouse *Slc7a11* (Hs. PT. 58.29117975), and mouse *Nqo1* (Hs. PT. 58.10871473) were purchased from Integrated DNA Technologies (Coralville, IA, USA). Expression data were normalized to 18S rRNA levels and presented as the fold difference in treated cells against untreated cells.

### 2.5. Cell Viability and Glutathione Level Assessment

Cell viability was determined by measuring ATP levels in viable cells using the CellTiter-Glo^®^ luminescent cell viability assay (Promega Corp., Madison, WI, USA). Cellular levels of reduced glutathione were measured using the GSH-Glo^TM^ Glutathione Assay (Promega Corp.), which detects the glutathione that reacts with glutathione *S*-transferase.

### 2.6. Western Blotting

Whole-cell lysate was prepared by lysing cells with a radioimmunoprecipitation assay (RIPA) buffer containing protease inhibitors (set III, Merck-Calbiochem, Tokyo, Japan). The lysates were heat-denatured with a reducing sample buffer, and the protein samples were analyzed by SDS-PAGE, followed by immunoblot analysis. An anti-tyrosinase antibody (T311) was obtained from Abcam (Cambridge, UK), and an anti-β-actin antibody (A2228) was obtained from Sigma-Aldrich (St Luis, MO, USA). Immune complexes were detected using anti-mouse IgG-HRP (NA9310) from GE Healthcare (Little Chalfont, UK) as a secondary antibody and SuperSignal™ West Femto Maximum Sensitivity Substrate (Thermo Fisher Scientific). The signal intensities were quantified with a LAS-4000 mini luminescent image analyzer (GE Healthcare).

### 2.7. Statistical Analysis

Statistical analyses of the results were performed using Welch’s two-tailed *t*-test for comparisons between two groups or using one-way ANOVA followed by Tukey’s post hoc test for comparisons among multiple groups.

## 3. Results

### 3.1. Knockdown of Nrf2 Enhances the Reduction in Viability of Melanoma Cells in a Tyrosinase-Dependent Manner Following Exposure to Leukoderma-Inducing Phenolic Compounds

**Figure 1:**
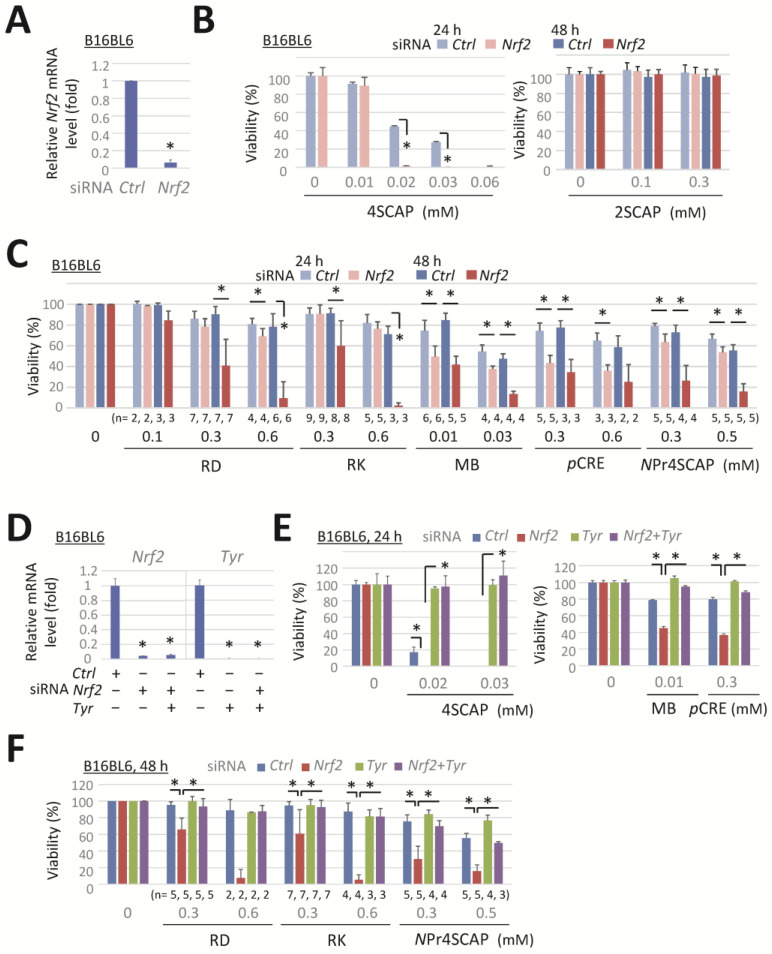

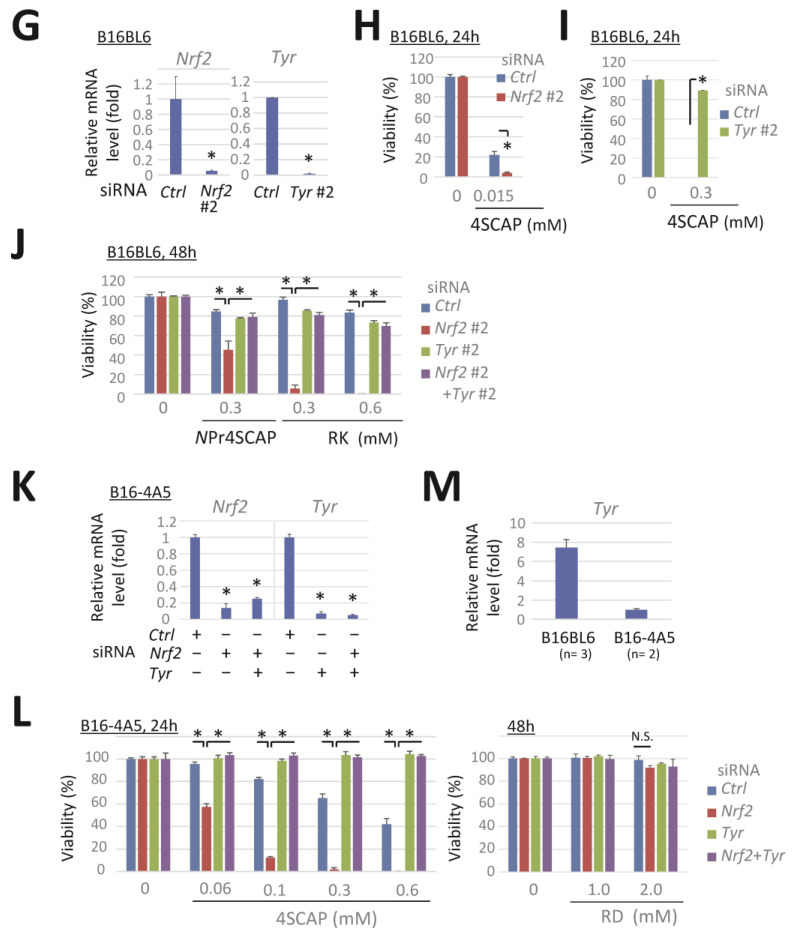
*Nrf2* knockdown enhances the reduction in viability of melanoma cells following their exposure to leukoderma-inducing phenolic compounds in a tyrosinase-dependent manner. B16BL6 melanoma cells were transfected with a negative control siRNA (*Ctrl*) or a siRNA directed against *Nrf2* (#1, MSS275988) or *Tyr* (#1, MSS212191) for 24 h. (**A**,**D**) Levels of *Nrf2* and *Tyr* mRNA following 24 h incubation in control medium. (**B**,**C**,**E**,**F**) The viability of cells following exposure to the indicated concentration of compounds for 24 h or 48 h. Data are presented as the means ± SD of independent experiments (n = 3 for (**A**) or as the number indicated for (**C**,**F**)) or the means ± SD (n = 3 wells) with similar results in more than two independent experiments (for (**B**,**D**,**E**)). *, *p* < 0.05, versus control siRNA-transfected cells (for (**A**,**D**)) or between respective values. B16BL6 melanoma cells (for **G**–**J**) were transfected with a siRNA directed against *Nrf2* (#2, MSS207018), *Tyr* (#2, MSS212190), or a negative control siRNA (*Ctrl*) for 24 h. B16-4A5 melanoma cells (for (**K**–**M**)) were transfected with *Nrf2* (#1) and *Tyr* (#1). (**G**,**K**,**M**). Levels of *Nrf2* or *Tyr* mRNA following 24 h incubation in control medium. (**H**,**I**,**J**,**L**) Viability of the cells following exposure to the indicated concentration of compounds for 24 h or 48 h. Data represent means ± SD (n = 3 wells). Similar results were obtained in two independent experiments (for (**H**,**I**,**J**,**L**)). For M, data represent the means ± SD of independent experiments (as the number indicated). *, *p* < 0.05, between respective values or versus control siRNA-transfected cells (for (**G**,**K**)).

We knocked down NRF2 to enhance the sensitivity of B16BL6 melanoma cells to the phenolic compounds. The transfection of B16BL6 cells with a specific siRNA against *Nrf2* (*Nfe2l2*) for 24 h effectively suppressed *Nrf2* mRNA expression (Figure 1A), which markedly enhanced the reduction in cell viability after 24 h of exposure to 4SCAP, MB, or pCRE (Figure 1B,C). RD, RK, and NPr4SCAP markedly reduced the viability of *Nrf2*-deficient cells after 48 h of exposure (Figure 1C), whereas no such reduction was observed in cells treated with 2SCAP (Figure 1B, right panel).

Subsequently, we examined the effect of tyrosinase knockdown on the enhancement of cytotoxicity induced by *Nrf2* knockdown to confirm whether *Nrf2* knockdown increases cell sensitivity to toxic tyrosinase metabolites. Transfection with a specific siRNA-targeting *Tyr*, *Nrf2*, and co-transfection with both siRNAs effectively suppressed target mRNA expression (Figure 1D). The reduction in cell viability following exposure to 4SCAP, MB, pCRE, or NPr4SCAP in negative-control siRNA-transfected cells was reversed by *Tyr* knockdown (Figure 1E). Moreover, the decrease in viability resulting from *Nrf2* knockdown following 24 h of exposure to 4SCAP, MB, or pCRE was prevented by the double knockdown of *Tyr* with *Nrf2*. The marked decline in viability resulting from *Nrf2* knockdown following 48 h exposure to RD, RK, or NPr4SCAP was also prevented by the simultaneous knockdown of *Tyr* and *Nrf2* (Figure 1F). These findings indicate that tyrosinase is essential for the enhancement of cytotoxicity by *Nrf2* knockdown and that *Nrf2* knockdown enhances cell sensitivity to the metabolites produced by tyrosinase.

This response was further evaluated with other siRNAs targeting *Nrf2* and *Tyr*. *Nrf2* (#2) and *Tyr* (#2) siRNAs effectively reduced the expression of the target mRNAs in B16BL6 melanoma cells (Figure 1G). Transfection with siRNA *Nrf2* (#2) enhanced the cytotoxicity of 4SCAP (Figure 1H) and elicited a pronounced reduction in viability following 48 h exposure to NPr4SCAP or RK (Figure 1J). Co-transfection with siRNA *Tyr* (#2) effectively prevented the observed reduction in cell viability (Figure 1I,J). These findings indicate that siRNAs *Nrf2* (#2) and *Tyr* (#2) elicited the same response as siRNAs *Nrf2* (#1) and *Tyr* (#1).

The enhanced cytotoxicity of phenolic compounds by *Nrf2* knockdown, which was reversed by *Tyr* knockdown, was further confirmed in a mouse melanoma cell line, B16-4A5. Transfection with siRNA *Nrf2* and/or siRNA *Tyr* resulted in a significant depletion of the target mRNA expression in B16-4A5 cells (Figure 1K). *Nrf2* knockdown significantly enhanced the cytotoxicity of 4SCAP, and this reduction in viability was completely prevented by the double knockdown of *Tyr* with *Nrf2*. However, the concentration of 4SCAP required to reduce the viability of B16-4A5 cells (Figure 1L, left panel) was approximately 10 times higher than that required for B16BL6 cells (Figure 1E, left panel). Up to 2 mM RD did not affect the viability of *Nrf2*-depleted B16-4A5 cells (Figure 1L, right panel). It is plausible that B16-4A5 cells did not produce sufficient amounts of RD metabolites to induce cytotoxicity. In order to verify this possibility, we examined the expression level of tyrosinase. As shown in Figure 1M, the mRNA expression of tyrosinase in B16-4A5 cells was approximately 7 times lower than that in B16BL6 cells.

### 3.2. Nrf2 Knockdown Reduces GSH, Slc7a11, and Nqo1 mRNA Levels and Effectively Prevents Their Induction by Phenolic Compounds

**Figure 2:**
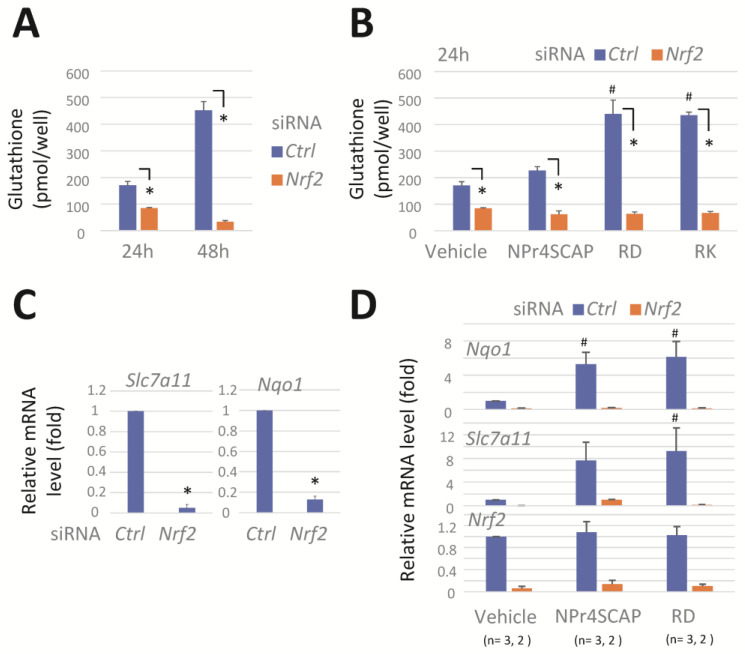
*Nrf2* knockdown reduces *GSH*, *Slc7a11,* and *Nqo1* mRNA levels and effectively prevents their induction by phenolic compounds. B16BL6 melanoma cells were transfected with a negative control siRNA (*Ctrl*) or a siRNA directed against *Nrf2* (#1) for 24 h. The cells were further incubated in the medium containing a vehicle or compounds (0.3 mM) for 24 h. (**A**,**B**) Cellular GSH levels. Data represent means ± SD (n = 3 wells). Similar results were obtained in two independent experiments. (**C**,**D**) Levels of *Slc7a11*, *Nqo1*, and *Nrf2* mRNA. Data represent means ± SD of independent experiments (n = 3 for (**C**), or as the number indicated for (**D**)). *, *p* < 0.05, versus control siRNA-transfected cells; #, *p* < 0.05, versus vehicle-treated cells.

NRF2 regulates the expression of genes involved in glutathione biosynthesis and their utilization in response to oxidative and electrophilic stress [32,33,34]. We evaluated the effect of *Nrf2* knockdown on cellular glutathione levels in B16BL6 cells. Transfection with siRNA *Nrf2* led to an approximately 50% to 90% reduction in glutathione levels after 24 h and 48 h, respectively (Figure 2A). Conversely, treatment with RD and RK increased the glutathione levels by more than 2-fold; however, this increase was potently suppressed by *Nrf2* knockdown (Figure 2B).

*Slc7a11* and *Nqo1* mRNA expressions were significantly downregulated by *Nrf2* knockdown in B16BL6 cells (Figure 2C). Exposure to RD and RK for 24 h markedly elevated the expression of *Slc7a11* and *Nqo1* mRNA. This elevation was effectively repressed by *Nrf2* knockdown (Figure 2D).

### 3.3. Slc7a11 Knockdown Enhances the Cytotoxicity Induced by RD, RK, and pCRE, Whereas Nqo1 Knockdown or Inhibition Is Almost Ineffective

**Figure 3:**
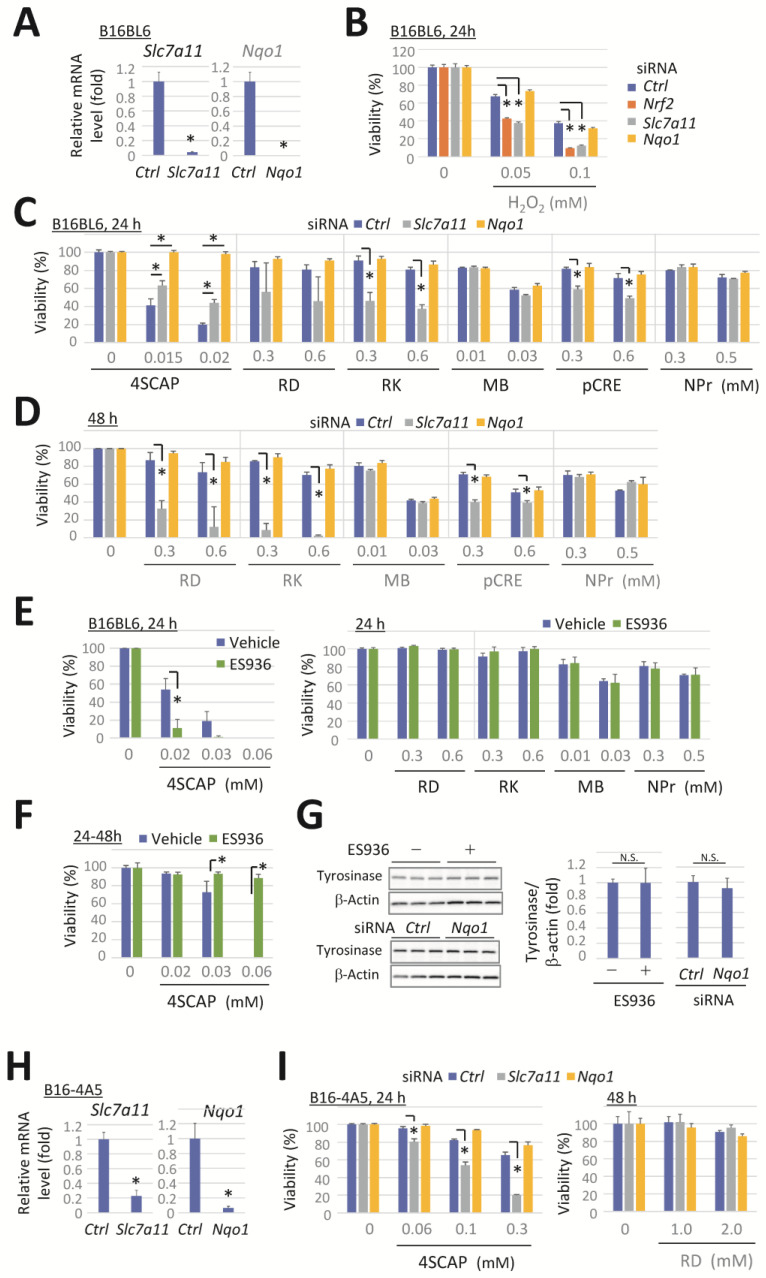
*Slc7a11* knockdown enhances the cytotoxicity induced by RD, RK, and pCRE, whereas *Nqo1* knockdown or inhibition is ineffective for most compounds. B16BL6 melanoma cells were transfected with a negative control siRNA (*Ctrl*) or a siRNA directed against *Slc7a11* or *Nqo1* for 24 h. (**A**) *Slc7a11* and *Nqo1* mRNA levels following 24 h incubation in a control medium. (**B**–**D**) The viability of cells following exposure to the indicated concentration of compounds for 24 h or 48 h. (**E**,**F**) The viability of B16BL6 cells after exposure to the indicated concentration of phenolic compounds in the presence or absence of ES936 (500 nM) for 24 h. (**F**) Cells were pretreated with a vehicle or ES936 for 24 h before their exposure to the compounds with/without ES936. (**G**) Western blots and quantitation of tyrosinase and the loading control of β-actin in cells at 48 h after *Nqo1*-siRNA transfection or 24 h after ES936 treatment. Data represent means ± SD (n = 3 wells) with similar results in two independent experiments (for (**A**–**F**)) or means ± SD of independent experiments (n = 4 for RD in panel C, D or n = 3 for 4SCAP in panel D). *, *p* < 0.05, versus *Ctrl*-siRNA-transfected cells (for (**A**)) or between respective values (for (**B**–**F**)). B16-4A5 melanoma cells were transfected with a negative control siRNA (*Ctrl*) or a siRNA directed against *Slc7a11* or *Nqo1* for 24 h. (**H**) Levels of *Slc7a11* and *Nqo1* mRNA following 24 h incubation in a control medium. (**I**) The viability of cells following exposure to the indicated concentration of compounds for 24 h or 48 h. Data represent means ± SD (n = 3 wells). Similar results were obtained in two independent experiments (for (**I**)). *, *p* < 0.05, versus *Ctrl*-siRNA-transfected cells.

We investigated the role of NRF2 target genes *Slc7a11* and *Nqo1* in protecting against the cytotoxicity of leukoderma-inducing phenols. The transfection of cells with siRNA specific for *Slc7a11* or *Nqo1* effectively suppressed the target mRNA expression (Figure 3A). The exposure of cells to H_2_O_2_ resulted in a concentration-dependent decrease in cell viability (Figure 3B). The knockdown of *Nrf2* or *Slc7a11* potentiated this decrease, indicating the role of *Nrf2* and *Slc7a11* for protection against oxidative stress. However, the knockdown of *Nqo1* had no effect.

Cells were exposed to leukoderma-inducing phenolic compounds at concentrations that significantly induced tyrosinase-dependent cytotoxicity in *Nrf2*-deficient cells (as shown in Figure 1E,F). *Slc7a11* knockdown markedly enhanced the reduction in cell viability after 24 h of exposure to RK or pCRE (Figure 3C), or after 48 h of exposure to RD (Figure 3D). In contrast, the cytotoxicity of 4SCAP was slightly reduced (Figure 3C), whereas *Slc7a11* knockdown had no effect on viability following exposure to the other compounds.

*Nqo1* knockdown suppressed the cytotoxicity of 4SCAP at the indicated concentrations, whereas no enhancement or suppression of the cytotoxicity of the other phenols was observed (Figure 3C,D). In contrast, ES936, a specific inhibitor of NQO1 [47,48], augmented the cytotoxic effect of 4SCAP when simultaneously exposed to cells with 4SCAP (Figure 3E, left panel), while the cytotoxicity of the other compounds was unaffected (Figure 3E, right panel). Conversely, the cytotoxicity of 4SCAP was suppressed by ES936 when the cells were pretreated with ES936 for 24 h (Figure 3F). The knockdown or inhibition of NQO1 has been reported to induce a reduction in tyrosinase expression in melanocytes [49]. Therefore, we conducted a Western blot analysis on the protein levels of tyrosinase in B16BL6 melanoma cells after 24 h of treatment with ES936 or 48 h following *Nqo1* knockdown; however, no effect was observed following either treatment (Figure 3G).

Additionally, the impact of *Slc7a11* and *Nqo1* knockdown on the cytotoxicity of leukoderma-inducing phenols was examined in B16-4A5 melanoma cells. The transfection of cells with siRNA specific for *Slc7a11* or *Nqo1* effectively suppressed the target mRNA expression (Figure 3H). Exposure to 4SCAP resulted in a concentration-dependent reduction in cell viability, which was enhanced by *Slc7a11* knockdown, whereas *Nqo1* knockdown had no effect. Furthermore, even after exposure to RD concentrations up to 2 mM, the knockdown of *Slc7a11* or *Nqo1* had no effect (Figure 3I).

## 4. Discussion

The metabolic activation of phenolic compounds by tyrosinase is a crucial step in the development of chemical leukoderma through the generation of highly reactive and toxic *o*-quinone metabolites [23]. In the present study, we developed a simple method to evaluate the metabolic activation of chemicals by analyzing tyrosinase-dependent cytotoxicity instead of direct metabolite analysis. We employed specific siRNA to deplete NRF2, which is a key transcriptional regulator of diverse cytoprotective genes [32,33,34], to overcome the insufficient sensitivity of B16BL6 cells to tyrosinase-produced metabolites [18]. As a result, all six phenolic compounds tested were detected in a tyrosinase-dependent manner, indicating an enhanced sensitivity to the *o*-quinone metabolites. A recently reported screening system for evaluating chemicals with leukoderma-inducing potencies focused on ER stress but did not include processes associated with the onset of leukoderma, such as tyrosinase dependency [27]. In contrast, this novel method of assessing the metabolic activation of chemicals by analyzing tyrosinase-dependent cytotoxicity in *Nrf2*-depleted melanoma cells is convenient and has superior sensitivity and specificity.

The cytotoxicity of RD and MB is enhanced by NRF2 knockdown in melanocytes [31,37]. Nonetheless, it is difficult to obtain melanocytes of the desired quality in sufficient quantities. In contrast, melanoma cells have the advantages of stable supply and proliferation capability. An increased sensitivity to the 4SCAP metabolites by the knockdown of *Nrf2* was observed in B16BL6 and B16-4A5 cells. However, the B16BL6 cells exhibited greater sensitivity than the B16-4A5 cells. The cytotoxic effect of 4SCAP was induced at concentrations 10 times lower in B16BL6 cells than in B16-4A5 cells. The high expression level (×7) of tyrosinase in B16BL6 cells compared with that in B16-4A5 cells may account for the observed difference in sensitivity to 4SCAP. The absence of enhanced RD cytotoxicity in B16-4A5 cells following *Nrf2*-depletion suggests that the tyrosinase levels were insufficient to produce toxic metabolites. In contrast, B16BL6 cells express elevated levels of hormonally stimulated adenylate cyclase [50], which may support the constant expression of high levels of tyrosinase via the cAMP–microphthalmia-associated transcription factor (MITF) system [51,52]. Therefore, B16BL6 cells may serve as a valuable cell model for evaluating the metabolic activation of phenolic compounds by tyrosinase.

The present study showed that the knockdown of *Slc7a11* in B16BL6 cells led to a pronounced increase in the cytotoxicity of RD, RK, and pCRE. These three compounds were shown to produce substantial quantities of cysteine/glutathione adducts with *o*-quinone metabolites in human tyrosinase-expressing 293T cells [18]. *Slc7a11* plays a crucial role in the cellular uptake of cystine and the maintenance of normal glutathione levels [32,45]. Notably, a mutation in *Slc7a11* causes a reduction in pheomelanin [45], which is formed by the addition of cysteine to *o*-dopaquinone, which is a tyrosinase metabolite of tyrosine. Furthermore, the depletion of cellular glutathione with an inhibitor of its synthesis causes an increase in RD cytotoxicity in melanocytes [31]. Thus, it is likely that *Slc7a11* knockdown decreases cysteine/glutathione adducts with *o*-quinone metabolites by reducing cellular cysteine and glutathione levels, thereby enhancing the cytotoxicity of phenols.

The NRF2-target NQO1 possesses catalytic activity to reduce quinones to hydroquinones, thereby facilitating the detoxification of quinones and the protection of cells from redox cycling and oxidative stress [40,41,42]. Conversely, certain anticancer agents have been shown to exert cytotoxic effects following a reduction of their *o*-quinone structure by NQO1 [42,43]. Therefore, we investigated the effect of *Nqo1* knockdown and its specific inhibition to determine whether NQO1 repressed or enhanced the cytotoxicity of *o*-quinone metabolites of leukoderma-inducing phenolic compounds. The knockdown or inhibition of *Nqo1* in B16BL6 cells did not affect the cytotoxicity of most compounds, in accordance with a report that the NQO1 inhibitor ES936 had no effect on RD cytotoxicity in this cell line [38]. However, only 4SCAP displayed a distinct response. ES936 augmented the cytotoxicity of 4SCAP when administered simultaneously. Conversely, the pretreatment of ES936 in cells resulted in the repression of 4SCAP cytotoxicity. Similarly, the exposure of 4SCAP to cells in which *Nqo1* was depleted with siRNA led to the complete repression of 4SCAP cytotoxicity.

This discrepancy can be explained as follows: NQO1 has the ability to protect cells from 4SCAP toxicity, as the overexpression of *NQO1* represses RD cytotoxicity [38]. However, this protective ability was lost after cells were pre-exposed to an NQO1 inhibitor or knocked down with *Nqo1* siRNA. Since the inhibition of tyrosinase with ES936 decreases tyrosinase protein levels in normal human melanocytes and the pigmentation of zebrafish embryos [49], it is plausible that tyrosinase is downregulated in B16BL6 cells following *Nqo1* knockdown or prolonged exposure to the inhibitor, thereby decreasing 4SCAP cytotoxicity. However, the Western blot analysis showed that tyrosinase protein levels were not affected by these treatments in the whole-cell lysates of B16BL6 cells. The effect of these treatments on tyrosinase activity remains unclear. Our findings indicate that *Nqo1* knockdown and inhibition have limited applicability in elucidating the role of NQO1. In contrast, conditional *NQO1* overexpression in B16BL6 cells, as reported by Okubo et al. for RD cytotoxicity [38], may serve as a valuable tool to understand the role of NQO1 and the mechanism by which leukoderma-induced phenols exert cytotoxic effects on melanogenic cells.

The transcription factor NRF2 is activated by ROS or electrophilic stress, such as *o*-quinones, and regulates a series of antioxidative enzymes and proteins [33]. In particular, numerous genes involved in glutathione synthesis and utilization participate in the protection of cells from oxidative and xenobiotic stress. In this study, the knockdown of *Nrf2* caused the significant depletion of glutathione in B16BL6 cells, and cellular glutathione was shown to be induced by phenolic compounds in a manner dependent on *Nrf2*. The KEAP1-NRF2 system plays a role in NRF2 activation in response to oxidative and xenobiotic stress [34]. KEAP1 serves as a sensor for ROS and electrophiles. Reactive cysteine residues in KEAP1 are directly modified, leading to NRF2 stabilization and the subsequent activation of transcription [34]. The metabolic activation of *p*-substituted phenols produces potent reactive metabolites, such as *o*-quinones [18], which may serve as potent inducers of NRF2-target genes. Indeed, RD and NPr4SCAP markedly induced *Slc7a11* and *Nqo1* mRNA expression.

In the present study, we have shown that NRF2 and its target SLC7A11 play a dominant role in protecting cells from toxic tyrosinase metabolites, which are a critical molecule in the pathogenesis of leukoderma. There is a large variation in sensitivity to RD among melanocytes of different origins [30], which could be attributed to differences in the NRF2 response. It is possible that impaired NRF2 response, as reported in vitiligo melanocytes [37], may contribute to the onset of leukoderma.

## 5. Conclusions

In the present study, we developed a method to evaluate the metabolic activation of chemicals by detecting the cytotoxicity induced by tyrosinase as an alternative to the direct analysis of metabolites [18]. Since B16BL6 melanoma cells in that study were insufficiently sensitive to tyrosinase metabolites, we knocked down the cytoprotective regulator NRF2 to enhance sensitivity. Consequently, all six leukoderma-inducing phenols exhibited tyrosinase-dependent cytotoxicity. Thus, the *Nrf2*-depleted B16BL6 cells may serve as useful models for evaluating the metabolic activation of chemicals by tyrosinase. This simple procedure can be used to screen a large number of test compounds. While the NRF2-target SLC7A11, a cystine transporter, was shown to suppress the cytotoxicity of certain compounds, the rapid inhibition of NQO1 highlighted its protective function in preventing 4SCAP-induced cytotoxicity. Further investigation of NRF2-targets may elucidate the mechanism through which the metabolic activation of leukoderma-inducing phenols elicits pathogenesis.

## Data Availability

The data are contained within the article and Supplementary Materials.

## Supplementary Materials

The following supporting information can be downloaded at https://www.mdpi.com/article/10.3390/biom15010114/s1. Figure S1: Original images of the Western blots for Figure 3G.
