# Supplementary material for: A Cell-Based Evaluation of the Tyrosinase-Mediated Metabolic Activation of Leukoderma-Inducing Phenols, II: The Depletion of Nrf2 Augments the Cytotoxic Effect Evoked by Tyrosinase in Melanogenic Cells
Source: Biomolecules. 2025 Jan 13;15(1):114. doi: 10.3390/biom15010114 (PMC11764042; doi:10.3390/biom15010114)

Supplementary Materials

A cell-based evaluation of tyrosinase-mediated metabolic activation of leukoderma-inducing phenols, II: Depletion of Nrf2 augments the cytotoxic effect evoked by tyrosinase in melanogenic cells

Tomoko Nishimaki-Mogami [1,*], Shosuke Ito [2], Kazumasa Wakamatsu [2,*], Takumi Akiyama [3], Norimasa Tamehiro [1] and Norihito Shibata [1]

Supplementary Material Contents

1. Original images

    Original images of western blots for Figure 3G

Original images for
Figure 3G upper panel

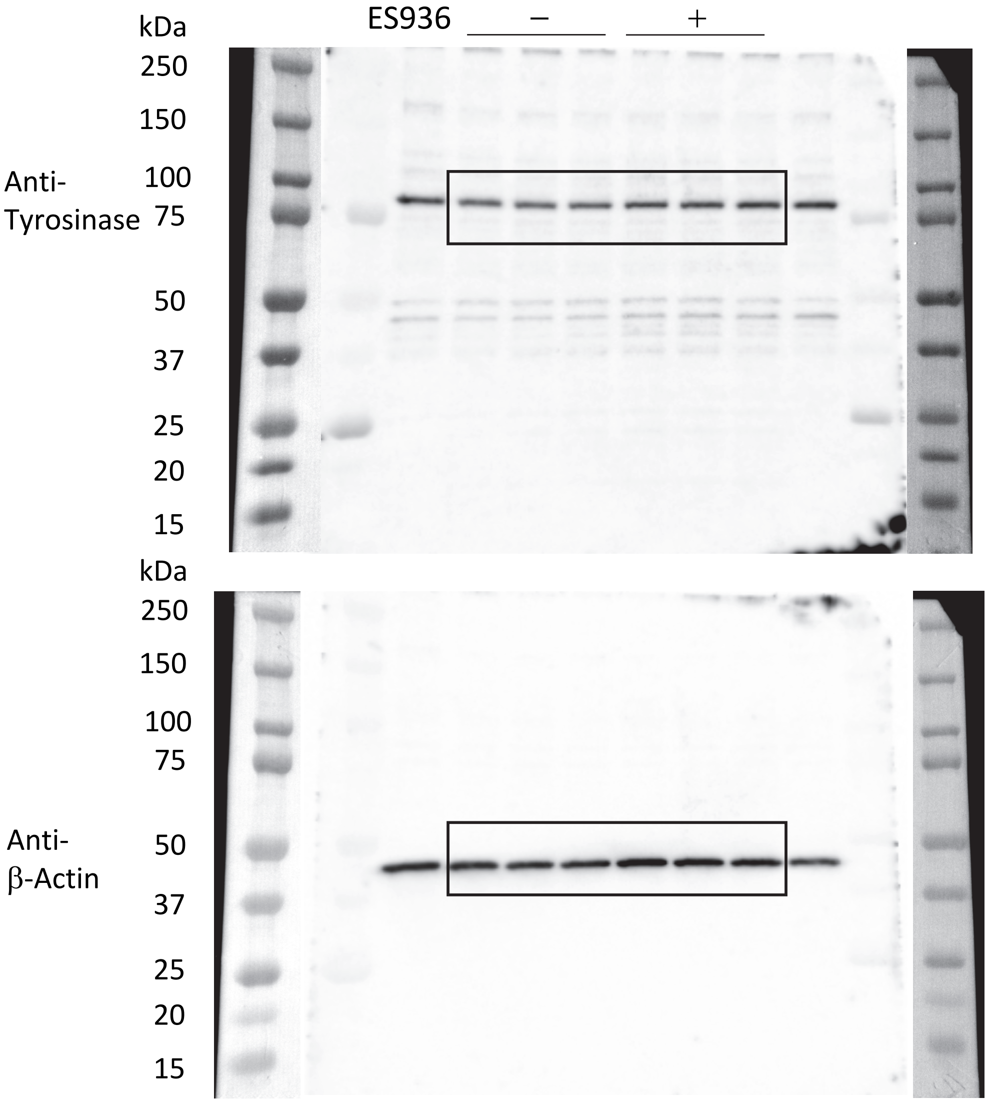

Original images for Figure 3G lower panel

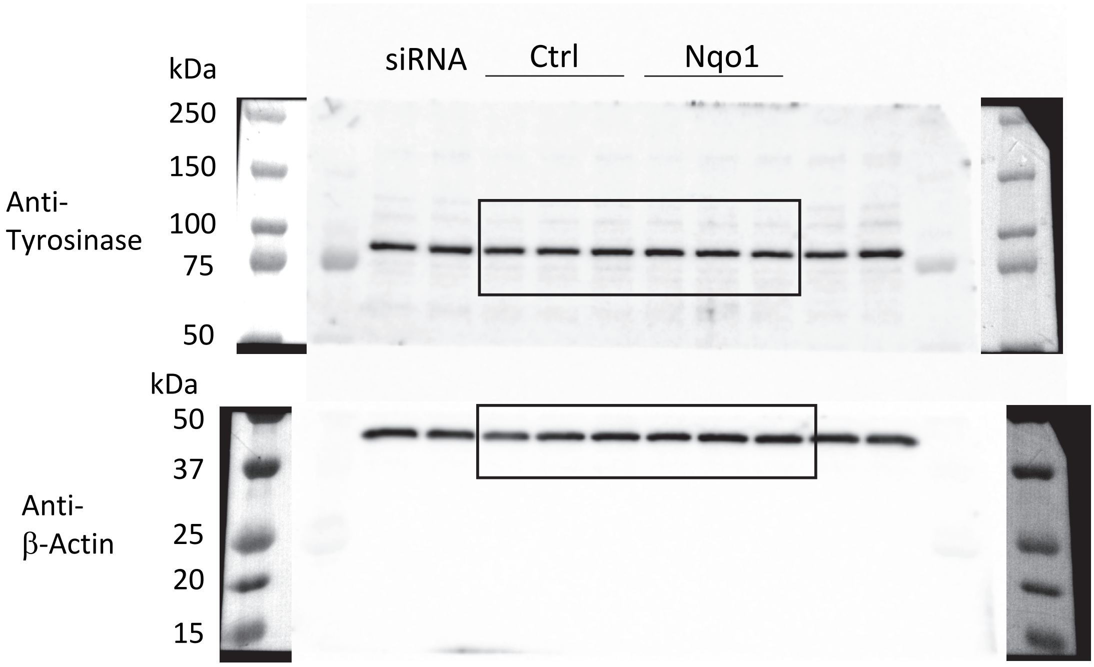

Supplement: Supplementary file 1 [file biomolecules-15-00114-s001.zip › biomolecules-3401240-supplementary.pdf]
